# Highly effective photoreduction of CO_2_ to CO promoted by integration of CdS with molecular redox catalysts through metal–organic frameworks†
†Electronic supplementary information (ESI) available. See DOI: 10.1039/c8sc02809e


**DOI:** 10.1039/c8sc02809e

**Published:** 2018-09-24

**Authors:** Chunjun Chen, Tianbin Wu, Haihong Wu, Huizhen Liu, Qingli Qian, Zhimin Liu, Guanying Yang, Buxing Han

**Affiliations:** a Beijing National Laboratory for Molecular Sciences , CAS Key Laboratory of Colloid and Interface and Thermodynamics , CAS Research/Education Center for Excellence in Molecular Sciences , Institute of Chemistry , Chinese Academy of Sciences , Beijing 100190 , P. R. China . Email: hanbx@iccas.ac.cn ; Email: wtb@iccas.ac.cn; b University of Chinese Academy of Sciences , Beijing 100049 , China; c Shanghai Key Laboratory of Green Chemistry and Chemical Processes , School of Chemistry and Molecular Engineering , East China Normal University , Shanghai 200062 , China

## Abstract

We propose a strategy to synthesize ternary CdS/UiO-bpy/Co composites by integration of CdS and molecular redox catalysts through metal–organic framework (MOF) UiO-bpy. The CdS/UiO-bpy/Co composites were very effective for photocatalytic conversion of CO_2_ to CO under visible light irradiation. The evolution rate of CO could reach 235 μmol g^–1^ h^–1^, which was a 10.2-fold improvement compared to that for the parent CdS, and selectivity for CO was 85%. The outstanding performance of the CdS/UiO-bpy/Co composites resulted from promoting the separation and migration of photo-induced charge carriers, enhancing the adsorption of CO_2_ molecules, and offering abundant active sites for CO_2_ reduction. As far as we know, this is the first study to integrate inorganic semiconductors and molecular redox catalysts through MOFs.

## Abstract 2

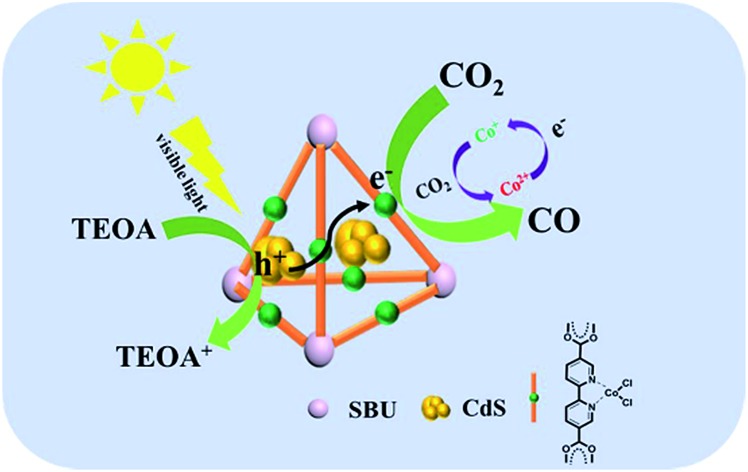
CdS/UiO-bpy/Co composites showed outstanding performance for photoreduction of CO_2_ to CO *via* integrating CdS with a Co complex through MOFs.

## Introduction

Global energy demand and climate change have underpinned broad interest in the sustainable reduction of CO_2_ into value added carbon products, such as CO, alcohols, and hydrocarbons. Photocatalytic reduction of CO_2_ is regarded as an essential and important technology for mitigation of the greenhouse effect and generation of renewable transportation fuels.^1^–^3^ Although great effort has been devoted to exploring various photocatalysts,^4^–^6^ the photocatalytic efficiency is far from satisfactory, mainly because of fast recombination of the photogenerated electron–hole pairs, low specific surface area and confined active sites.^7^–^9^ Increasing the CO_2_ adsorption ability of photocatalysts is an effective strategy to improve the CO_2_ conversion efficiency.^10^,^11^ Due to their large surface area and well-ordered porous structure, metal–organic frameworks (MOFs) are considered to be promising candidates for gas adsorption, and can facilitate CO_2_ adsorption.^12^,^13^ Nevertheless, the performance of MOFs in photocatalytic reduction of CO_2_ is obstructed by the low efficiency of exciton generation and charge separation in MOFs, as well as inconsistencies between catalytic and adsorption sites.^14^,^15^

Cadmium sulfide (CdS), with an excellent visible-light response and appropriately positioned conduction band, has been investigated extensively as a photocatalyst for photocatalytic reduction of CO_2_, whose applications have unfortunately been limited due to the fast recombination of photogenerated electron–hole pairs, the serious photocorrosion, the low CO_2_ adsorption, and the lack of catalytic sites.^16^–^18^ It has been reported that the combination of CdS and MOFs can help to surmount their respective weak points by increasing the visible-light response and facilitating CO_2_ adsorption.^19^ However, it is difficult for CdS/MOF composites to achieve high photocatalytic performance for CO_2_ reduction due to the large size of CdS and lack of catalytic sites.^20^

Some molecular redox catalysts containing metals such as Re, Co, Ni, and Fe are capable of reducing CO_2_ to CO photochemically.^21^,^22^ However, they need to be fed with electrons by a photosensitizer. So far, a number of studies have been proposed to utilize inorganic semiconductors as photosensitizers,^23^–^25^ but the interaction between inorganic semiconductors and complexes is very poor, leading to difficulty in cycling experiments. It was reported that bipyridine cobalt complexes can efficiently promote the photoreduction of CO_2_ to CO when combined with these inorganic semiconductors.^26^ However, bipyridine cobalt complexes can dissolve in the solution, which is unfavorable for recovery. Although a few studies have been reported on combining MOFs and molecular redox catalysts for photocatalytic reduction of CO_2_, either precious metals or complex ligands were used.^27^,^28^ UiO-bpy MOFs, with exceptional CO_2_ adsorption capacity and stability,^29^ have been constructed from linear 2,2′-bipyridine-5,5′-dicarboxylate (bpydc) bridging ligands and Zr_6_(μ_3_-O)_4_(μ_3_-OH)_4_ SBUs. Although the bridging ligands of bpydc can coordinate with some transition metal (*e.g.* Co^2+^, Cu^2+^ and Ni^2+^), the CO_2_ photoreduction is also obstructed by the low efficiency of visible light exciton generation.

In this work, we designed ternary CdS/UiO-bpy/Co composites. The inorganic semiconductors and the molecular redox catalysts were integrated through UiO-bpy. The CdS/UiO-bpy/Co composites showed outstanding performance for photocatalytic conversion of CO_2_ to CO under visible light irradiation. To the best of our knowledge, there is no report on the combination of inorganic semiconductors and molecular redox catalysts through MOFs for photoreduction of CO_2_.

## Results and discussion

**Fig. 1:**
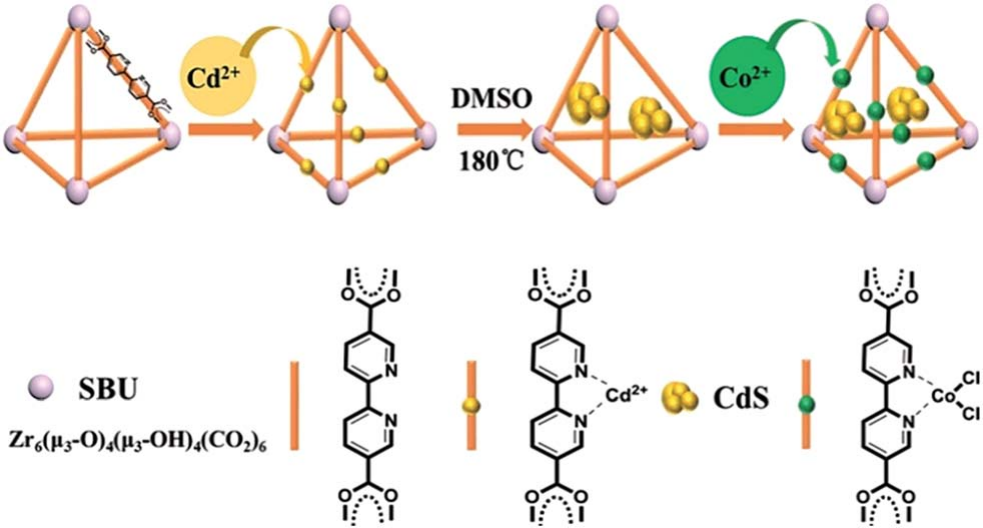
The route for the synthesis of the CdS/UiO-bpy/Co composites.

The method proposed to synthesize the ternary CdS/UiO-bpy/Co composites is shown schematically in Fig. 1. UiO-bpy was firstly prepared from ZrCl_4_ and bpydc following a reported method.^30^ Then, the vacuum-activated sample of UiO-bpy was treated with Cd(CH_3_COO)_2_·2H_2_O and dimethylsulfoxide (DMSO) at 180 °C for 12 h to form CdS/UiO-bpy. Because the bpydc bridging ligands can coordinate with Cd^2+^ in DMSO, and the Cd^2+^ can slowly react with DMSO to form CdS,^31^ small CdS nanoparticles were generated. The synthesized CdS/UiO-bpy samples and cobalt chloride were added into tetrahydrofuran (THF) solvent at room temperature and stirred for 12 h. Finally, the CoCl_2_ was integrated into CdS/UiO-bpy by coordinating with bpydc and the ternary CdS/UiO-bpy/Co composites were obtained in this way.

The obtained CdS, UiO-bpy, CdS/UiO-bpy and CdS/UiO-bpy/Co composites were characterized by X-ray diffraction (XRD), as shown in Fig. S1.† For these samples, a group of strong diffraction peaks before 20° matched with the simulated UiO-bpy signals,^32^ proving that CdS and Co loading did not change the structure of UiO-bpy. In addition, the three diffraction peaks with 2*θ* values of 26.5°, 44.0, and 52.1° are related to the (111), (220), and (311) crystal planes of hawleyite CdS, respectively. The contents of CdS and Co^2+^ were determined by ICP-AES (VISTA-MPX) and the results are shown in Table S1.† The loading amount of Co in the CdS/UiO-bpy/Co composites was determined to be 6.18 wt%, indicating that 50.8% of the bpy units were metalated with Co in the UiO-bpy framework.

**Fig. 2:**
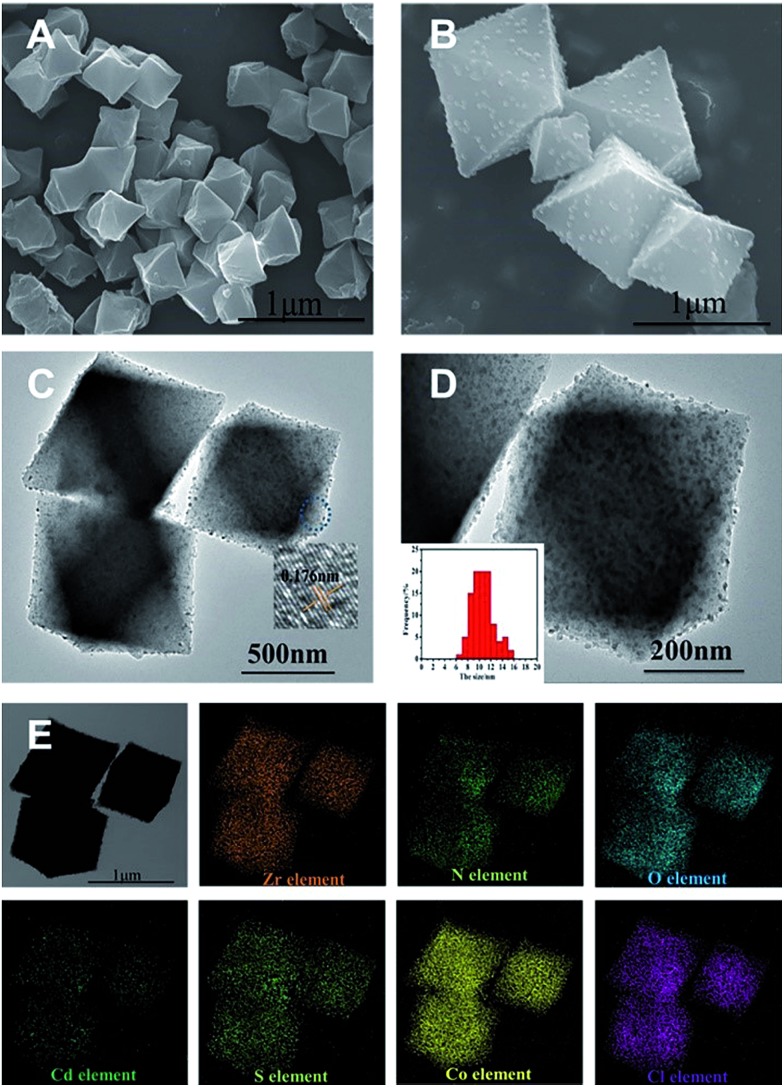
(A) SEM image of UiO-bpy. (B) SEM image of CdS/UiO-bpy/Co. (C) TEM image of CdS/UiO-bpy/Co and the high-resolution TEM image of the selected area. (D) The high-magnification TEM image of CdS/UiO-bpy/Co and the particle size distribution of CdS. (E) EDX mapping of CdS/UiO-bpy/Co.

The scanning electron microscopy (SEM), transmission electron microscopy (TEM), and high-resolution TEM (HR-TEM) images of the as-synthesized UiO-bpy and CdS/UiO-bpy/Co composites are shown in Fig. 2. We can observe that the UiO-bpy is a regular octahedron, and the morphology of UiO-bpy was retained after the UiO-bpy was loaded with CdS and Co, as shown in Fig. 2A and B. It is found that the CdS nanoparticles with a diameter of about 10 nm were well dispersed on the UiO-68 framework in the CdS/UiO-bpy/Co composites, as shown in Fig. 2C and D. The lattice fringes of individual CdS particles with an interplanar spacing of ≈0.176 nm can be assigned to the (311) lattice plane of hawleyite (Fig. 2C), which is in good agreement with XRD analysis (Fig. S1†). Energy dispersive X-ray (EDX) elemental mapping of the CdS/UiO-bpy/Co composites in Fig. 2E shows that Cd, S, Co and Cl elements were dispersed uniformly in the composites.

The CdS, UiO-bpy, CdS/UiO-bpy and CdS/UiO-bpy/Co composites were characterized using the N_2_ adsorption–desorption method. As shown in Fig. S2,† pure UiO-bpy had a total surface area (*S*_BET_) of 1323 m^2^ g^–1^, while the *S*_BET_ value based on the total weight of CdS/UiO-bpy was 52.6 m^2^ g^–1^. The decrease in *S*_BET_ after incorporation of CdS is due to the fact that non-porous CdS nanoparticles blocked some pores of UiO-bpy. The *S*_BET_ of the CdS/UiO-bpy/Co composites decreased to 25.5 m^2^ g^–1^ due to high metal loading and the potential blocking of pore entrances by the sterically demanding CoCl_2_. Despite the fact that the BET surface area of the CdS/UiO-bpy/Co composites was smaller than that of pure CdS, the CO_2_ adsorption amount was larger than that of pure CdS, as shown in Fig. S3,† which indicates that the UiO-bpy can facilitate CO_2_ adsorption.

The size of CdS in the CdS/UiO-bpy was smaller than that of the pure CdS, as can be seen from Fig. S4,† mainly resulting from the coordination of bpydc with Cd^2+^. In order to elucidate the role of bpydc, we replaced the bpydc with biphenyl-4,4′-dicarboxylic acid. Similar to bpydc, biphenyl-4,4′-dicarboxylic acid can also react with ZrCl_4_ to produce UiO-67, and the morphology of UiO-67 was similar to that of the UiO-bpy (Fig. S5a†). The preparation conditions for CdS/UiO-67 were the same as those for CdS/UiO-bpy. However, as shown in Fig. S5b,† the size of CdS particles in CdS/UiO-67 was about 50 nm, which was larger than that in CdS/UiO-bpy (10 nm, Fig. 2), mainly because biphenyl-4,4′-dicarboxylic acid does not coordinate with Cd^2+^. So we can conclude that the bridging ligands of bpydc played a key role in obtaining small size CdS nanoparticles. Furthermore, we also found that the amount of cadmium acetate had an important effect on the size of CdS, as shown in Fig. S6.†

**Fig. 3:**
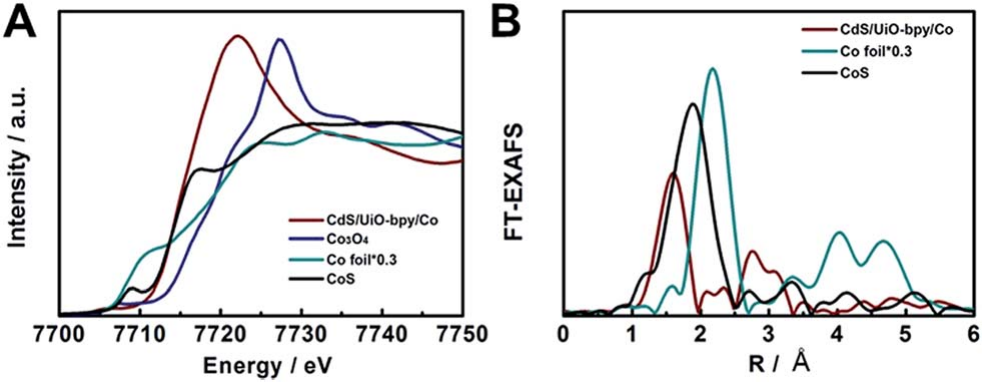
(A) XANES spectra at the Co K-edge. (B) Fourier-transformed Co K-edge EXAFS spectra.

In order to elucidate the local coordination environment of the Co atom in the CdS/UiO-bpy/Co composites, X-ray absorption near-edge structure (XANES) spectra and Fourier-transformed Co K-edge extended X-ray absorption fine structure (EXAFS) spectra were recorded. As shown in Fig. 3A, the absorption edge position of the CdS/UiO-bpy/Co composites is located between that of Co foil and Co_3_O_4_ and is close to that of CoS, suggesting that the Co atom carries positive charge and the valence state of Co is between +1 and +3. As shown in the Fourier transform of the EXAFS spectrum of CdS/UiO-bpy/Co composites (Fig. 3B), the peaks at around 1.6 Å can be attributed to the Co–N bond and the Co–Cl bond. The quantitative coordination configuration of the Co atom can be obtained by EXAFS fitting (Fig. S7 and Table S2†). Both the coordination numbers of Co–N and Co–Cl are 2. These results indicate that the coordination structure of the Co atom is consistent with the schematic diagram in Fig. 1. Diffuse reflectance UV/Vis spectroscopy and X-ray photoelectron spectroscopy (XPS) were also used to elucidate the local coordination environment of the Co atom in the CdS/UiO-bpy/Co composites. A typical absorption band in the region of 600–800 nm corresponding to the Co complex in the UV/vis spectrum was observed, as shown in Fig. S8.† It was also confirmed that the absorption properties are similar to those of the corresponding homogeneous complex Co-bpy, indicating that the Co atoms were coordinated with the ligands of bpydc, which was further supported by the XPS survey spectra in Fig. S9.† The N 1s XPS spectra of CdS/UiO-bpy shifted to higher binding energies by 0.16 eV after the Co atoms were loaded into the CdS/UiO-bpy, suggesting that the surface chemical states of N atoms were changed by the loaded Co atoms, due to the interaction between the N atoms and Co atoms in the CdS/UiO-bpy/Co composites. XPS spectra of the Co 2p and Cl 2p also indicated the introduction of Co and Cl elements into the framework (Fig. S10†).

**Fig. 4:**
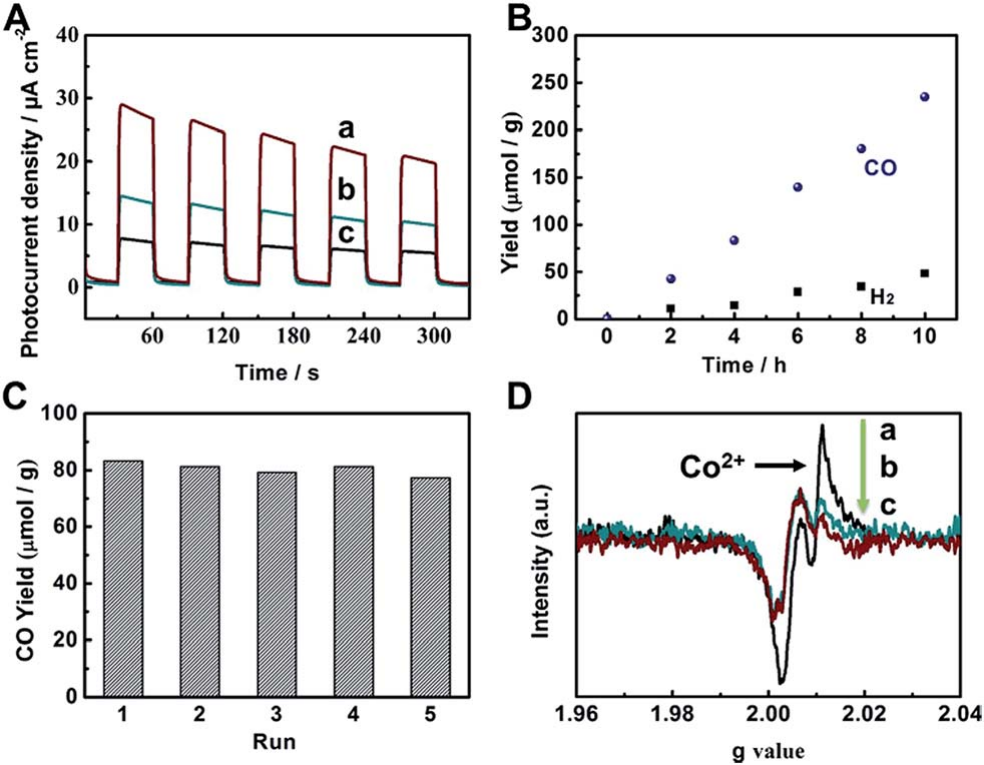
(A) Transient photocurrent responses under visible light irradiation (*λ* ≥ 420 nm): CdS/UiO-bpy/Co (a), CdS/UiO-bpy (b), and CdS (c). (B) Yields of CO and H_2_ over CdS/UiO-bpy/Co. (C) Reusability of CdS/UiO-bpy/Co with a reaction time of 4 h for each cycle. (D) EPR spectra of CdS/UiO-bpy/Co under different conditions. Key: without light and N_2_ (a), with light and CO_2_ (b), with light and N_2_ (c).

To evaluate the photocatalytic applications of CdS, UiO-bpy, CdS/UiO-bpy, and CdS/UiO-bpy/Co composites, their optical absorption properties were investigated by UV-vis diffuse reflectance spectroscopy, as shown in Fig. S8.† It is shown that the UiO-bpy can only absorb ultraviolet light. However, CdS, CdS/UiO-bpy and CdS/UiO-bpy/Co composites exhibited strong optical absorption extending to the visible light region, suggesting that these materials can be excited by visible light irradiation to generate electron–hole pairs for redox reactions. To gain a deeper understanding of the efficacy of photoexcited charge separation, the photo-current responses of CdS, CdS/UiO-bpy and CdS/UiO-bpy/Co composites were tested, as shown in Fig. 4A. The transient photocurrent results provide clear evidence for electron transfer within these catalysts. It is clear that the photocurrent intensity of CdS/UiO-bpy/Co was higher than that of both CdS and CdS/UiO-bpy under visible light irradiation, indicating more effective charge separation in the CdS/UiO-bpy/Co composites.

The catalytic behaviors of the CdS/UiO-bpy/Co composites were examined for the photochemical reduction of CO_2_ and compared with those of CdS and CdS/UiO-bpy. The CO_2_ reduction was conducted under visible light irradiation, using triethanolamine (TEOA) as an electron donor and acetonitrile as the solvent. As shown in Fig. 4B, the amount of CO generated increased almost linearly with irradiation time. The CdS/UiO-bpy/Co composites showed a high CO evolution rate of 235 μmol g^–1^ h^–1^ under light illumination for 10 hours, which was higher than that of CdS (CO, 23 μmol g^–1^ h^–1^) and CdS/UiO-bpy (CO, 0 μmol g^–1^ h^–1^), as shown in Table 1. The turnover number of CO formation using the CdS/UiO-bpy/Co composites was 2.24. The CO evolution rate is 195 μmol g^–1^ h^–1^ for the CdS/UiO-bpy/Co composites under ambient pressure of CO_2_ (Table 1). And the CO evolution rate was 110 μmol g^–1^ h^–1^ for CdS with the aid of Co-bpy as a cocatalyst, which was lower than that of the CdS/UiO-bpy/Co composites (Table 1). These results reveal that CdS/UiO-bpy/Co composites have comparable activity for CO_2_ reduction compared with previously reported catalysts under comparative conditions, as can be seen from Table S3.† The yield and the morphology of the catalyst had no obvious change after five cycles, as shown in Fig. 4C and S11,† indicating the excellent stability of the CdS/UiO-bpy/Co composites. Our experiments showed that in the absence of photocatalysts or light illumination, no products were detected in the reaction, implying that CO was generated by the photocatalytic reduction of CO_2_ on the CdS/UiO-bpy/Co composites, as shown in Table 1. In order to confirm that the reaction is induced by the light absorption of CdS, the action spectrum for the photocatalytic reaction was determined, as shown in Fig. S12.† Furthermore, the calculated apparent quantum yield of the CdS/UiO-bpy/Co composites at 420 nm was 0.65%, which was higher than that of CdS (0.063%) and CdS@Co-bpy (0.302%). Comparable AQY was previously reported for CO_2_ to CO conversion with a CdS-[Ni(terpyS)_2_]^2+^ hybrid catalyst (0.28 ± 0.04%)^33^ and CO_2_ to formate conversion with a Ru–Ag–TaON hybrid catalyst (0.48%).^34^ It is also found that higher efficiencies can be achieved by using a RuP/C_3_N_4_ hybrid catalyst with a suitable solvent of *N*,*N*-dimethylacetamide (5.7%).^35^

**Table 1:** Research on conditions and control experiments^*a*^

Entry	CO (μmol g^–1^ h^–1^)	H_2_ (μmol g^–1^ h^–1^)	Selectivity of CO^*b*^ (%)	AQY_CO_^*c*^ (%)
1	235	41	85	0.65
2^*d*^	23	248	8	0.063
3^*e*^	nd	275	0	—
4^*f*^	nd	580	0	—
5^*g*^	nd	nd	—	—
6^*h*^	nd	nd	—	—
7^*i*^	110	132	45	0.302
8^*j*^	195	45	81	0.54

^*a*^Reaction conditions: CdS/UiO-bpy/Co composites (10 mg), CH_3_CN (3 mL), TEOA (0.5 mL), CO_2_ (0.8 MPa), 300 W Xe lamp, *λ* ≥ 420 nm, 10 h.

^*b*^Selectivity of CO = *n*CO/(*n*CO + *n*H_2_).

^*c*^Apparent Quantum Yield (AQY): AQY_CO_ = 2 × the number of evolved CO molecules/the number of incident photons.

^*d*^Using CdS instead of CdS/UiO-bpy/Co composites.

^*e*^Using CdS/UiO-bpy instead of CdS/UiO-bpy/Co composites.

^*f*^Using CdS/UiO-bpy/Ni composites instead of CdS/UiO-bpy/Co composites.

^*g*^In the dark.

^*h*^Using N_2_ instead of CO_2_. nd: not detected.

^*i*^Using CdS and Co-bpy instead of CdS/UiO-bpy/Co composites.

^*j*^The pressure of CO_2_ was 0.1 MPa.

To further validate the source of the generated CO, an isotopic experiment using ^13^CO_2_ as a substrate was performed under identical photocatalytic reaction conditions, and the products were analyzed by gas chromatography and mass spectrometry. As shown in Fig. S13,† the peak at *m*/*z* = 29 could be assigned to ^13^CO, indicating that the carbon source of CO was the CO_2_ used. When the CO_2_ was replaced by N_2_, no detectable products were formed, as shown in Table 1.

In order to clarify the role of Co in the CdS/UiO-bpy/Co composites, we also loaded Ni atoms into the preformed CdS/UiO-bpy composites to form ternary CdS/UiO-bpy/Ni composites, which were similar to CdS/UiO-bpy/Co composites, and the results are shown in Fig. S14.† The CdS/UiO-bpy/Ni composites displayed 2.1-fold higher photocatalytic activity for H_2_ evolution compared with CdS/UiO-bpy, but no CO was generated, as shown in Table 1. This indicated that the Co in the composites was crucial for the photoreduction of CO_2_ to CO. Electron paramagnetic resonance (EPR) spectroscopy was used to further study the function of Co atoms during the CO_2_ photoreduction process. As shown in Fig. 4D(a), a strong signal for high-spin state Co^2+^ was observed for the CdS/UiO-bpy/Co composite without irradiation in a nitrogen atmosphere.^36^ However, the peak intensity of Co^2+^ was greatly weakened with light irradiation, which clearly indicates that the Co valence states transformed from high-spin state Co^2+^ to Co^+^ in a low-spin state (Fig. 4D(c)). The EPR signal corresponding to Co^2+^ was enhanced when CO_2_ was introduced into the irradiated CdS/UiO-bpy/Co composites, implying that some of the Co^+^ was oxidized back to Co^2+^ species during the CO_2_ photoreduction process (Fig. 4D(b)).^37^ This kinetic behavior also indicates that photoexcited electrons are transferred to the Co center,^38^,^39^ and the photoreduction of CO_2_ was enhanced by the valence transformation of the Co.

## Conclusions

In conclusion, we demonstrate the design and fabrication of ternary CdS/UiO-bpy/Co composites, which combined inorganic semiconductors with molecular redox catalysts through MOFs using the function of the ligands. CdS nanoparticles of small size were obtained due to the presence of bridging ligands of bpydc. The ternary CdS/UiO-bpy/Co composites exhibited very high efficiency for photoreduction of CO_2_, affording CO as the sole carbonaceous product with an evolution rate of 235 μmol g^–1^ h^–1^, and the selectivity was over 85%. We anticipate that the implantation strategy, combining inorganic semiconductors with molecular redox catalysts through MOFs, will be applicable for designing many other efficient photocatalysts for CO_2_ reduction.

## Conflicts of interest

There are no conflicts to declare.

## Supplementary Material

Supplementary information
