# Supplementary material for: Highly effective photoreduction of CO2 to CO promoted by integration of CdS with molecular redox catalysts through metal–organic frameworks
Source: Chem Sci. 2018 Sep 24;9(47):8890–4. doi: 10.1039/c8sc02809e (PMC6296294; doi:10.1039/c8sc02809e)
Supplement: Supplementary file 1 [file SC-009-C8SC02809E-s001.pdf]

Electronic Supplementary Material (ESI) for Chemical Science.
This journal is © The Royal Society of Chemistry 2018

Supporting Information

Highly effective photoreduction of CO_2 to CO promoted by integration of CdS with molecular redox catalysts through metal–organic frameworks

Chunjun Chen,[a,b] Tianbin Wu,[a]* Haihong Wu,[c] Huizhen Liu,[a,b] Qingli Qian,[a] Zhimin Liu,[a,b] Guanying Yang,[a] Buxing Han[a,b]*

a. Beijing National Laboratory for Molecular Sciences, Key Laboratory of Colloid and Interface and Thermodynamics, Institute of Chemistry, Chinese Academy of Sciences, Beijing 100190, China

b. School of Chemistry and Chemical Engineering, University of Chinese Academy of Sciences, Beijing 100049, China

c. Shanghai Key Laboratory of Green Chemistry and Chemical Processes, School of Chemistry and Molecular Engineering, East China Normal University, Shanghai 200062, China

Experimental Section

Materials. $Cd(CH_3COO)_2 \cdot 2H_2O$ (A. R. grade), $CoCl_2 \cdot 6H_2O$ (A. R. grade), $NiCl_2 \cdot 6H_2O$ (A. R. grade), N,N-Dimethylformamide (DMF, A. R. grade), ethanol (A. R. grade), acetone (A. R. grade), dimethyl sulfoxide (A. R. grade), tetrahydrofuran (A. R. grade) were provided by Sinopharm Chemical Reagent Co., Ltd. 2,2'-Bipyridine-5,5'-dicarboxylate (purity>99%), tris(2-hydroxyethyl)amine (TEOA, purity>99%), biphenyl-4,4'-dicarboxylic acid (purity>99%), Zirconium tetrachloride (purity>99%) were obtained from J&K Scientific Ltd. Formic acid (purity>96%) was provided by Alfa Aesar China (Tianjin) Co., Ltd.

Synthesis of UiO-bpy. The method reported for synthesis of UiO-bpy was used.[s1] In the experiment, $ZrCl_4$ (73.5 mg, 0.318 mmol), 2,2-bipyridine-5,5-dicarboxylic acid (bpydc, 78 mg, 0.312 mmol), HCO_2H (3 mL), and DMF (30 mL) were charged into a vial and heated at 120 °C for 24 h. The resulting solid was collected, washed with DMF and THF, and then dried overnight at 120 °C under vacuum to obtain white powdery product UiO-bpy. The UiO-67 was prepared using similar method except that the bpydc was replaced by the biphenyl-4, 4'-dicarboxylic acid (75 mg, 0.312 mmol).

Synthesis of CdS/UiO-bpy. 0.3 g of UiO-bpy, 30 mg of $Cd(CH_3COO)_2 \cdot 2H_2O$ and 80 ml of DMSO were introduced into a 100 mL flask and stirred for 1 h. Then the mixed solution was poured into a Teflon-lined stainless steel autoclave with 100 mL volume, and maintained at 180 °C for 10 h. After the system was naturally cooled down to room temperature, the obtained yellow suspension was washed by water and ethanol for 6 times and dried overnight at 60 °C under vacuum. The content of CdS nanoparticles was controlled by changing amount of $Cd(CH_3COO)_2 \cdot 2H_2O$ with fixed amount of UiO-bpy (0.3 g). Pure CdS was also prepared via a similar procedure but without the addition of UiO-bpy. The CdS/UiO-67 was also prepared via a similar procedure except that the UiO-bpy was replaced by the UiO-67.

Synthesis of CdS/UiO-bpy/Co. In a vial, $CoCl_2 \cdot 6H_2O$ (71.0 mg, 0.30 mmol) was dissolved in 20 mL of THF. CdS/UiO-bpy (100 mg) was added to the $CoCl_2$

solution. The resulting suspension was slowly stirred overnight at room temperature. The green solid was then centrifuged and washed with THF for 6 times, and dried overnight at 60 °C under vacuum to obtain the CdS/UiO-bpy/Co. The CdS/UiO-68/Ni was also prepared via a similar procedure except that the $CoCl_2·6H_2O$ was replaced by the $NiCl_2·6H_2O$.

Synthesis of Co-bpy. In a vial, 2, 2′-bipyridine (156 mg) and $CoCl_2$ (129 mg) were added to acetonitrile (30 mL), and the mixture was heated at 313 K in air. The green powder was collected by filtration and dried under vacuum.

Materials characterizations. The microstructures of the catalysts were characterized by scanning electron microscope (SEM, HITACHI S-4800) and transmission electron microscopy (TEM, JEOL JEM-2100F) equipped with EDS. X-ray photoelectron spectroscopy (XPS) study was carried out on the Thermo Scientific ESCALab 250Xi using a 200W Al-Kα radiation. In the analysis chamber, the base pressure was about 3×10^{-10} mbar. Typically, the hydrocarbon C1s line at 284.8 eV from adventitious carbon was used for energy referencing. X-Ray diffraction (XRD) analysis of the samples were performed on a Rigaku D/max-2500 X-ray diffractometer with Cu-Kα radiation (y = 0.15406 nm) and the scan speed was 5 ^{o}/min. The N_2 adsorption/desorption isotherms were determined using a Micromeritics ASAP 2020 sorptometer operated at 77 K, and then BET surface areas and pore volumes were obtained. The adsorption isotherms of CO_2 were determined at 298 K in the pressure range of 0-1 atm on a TriStar II 3020 device. The CdS and Co loadings in the catalysts were determined by ICP-AES method (VISTA-MPX). The UV-Vis absorption was measured with a UV-visible spectrophotometer (Shimadzu, UV-2600) using $BaSO_4$ as the reflectance standard reference. EPR measurements of sample were carried out at room temperature on a JEOL E-500 by addition of sample to CH_3CN and TEOA solution.

The photoelectrochemical (PEC) properties. The photoelectrochemical (PEC) properties of the samples were carried out on the electrochemical workstation (CHI 6005E). The experiment was conducted in a single cell with a three electrodes configuration, which was consisted of working electrode, a platinum foil as counter

electrode, and Ag/AgCl (saturated KCl solution) as reference electrode. The working electrode was fabricated by dropcasting an ethanol suspension of photocatalyst samples on indium-tin oxide (ITO) glasses slide within an active area of 1 cm^2. Na_2SO_4 aqueous solution (0.5 M) served as the electrolyte. The light illumination was supplied by a 300 W Xe arc lamp equipped with a 420 nm cut-off filter in the PEC measurements. An applied bias of 0.2 V was used to measure the transient photocurrent response.

Apparent Quantum Yield (AQY) calculations. The AQY for CO evolution for the CdS and CdS/UiO-bpy/Co composites was measured using the standard experimental setup. The system was irradiated by 300 W Xe lamp equipped with a 420 nm optical band-pass filter and subjected to react for 10 h. The amount of CO evolved in the duration of 10 h for CdS, CdS@Co-bpy and CdS/UiO-bpy/Co composites was 0.98, 4.7 and 10.1 μmol respectively. The average intensity of irradiation was determined to be 5 mW/cm^{-2} by a CEL-NP2000 spectrometer. The irradiation area was measured to be 4.91 cm^2. Finally, the AQY was then estimated as follows:

$$QE = \frac{2 \times the\ number\ of\ evolved\ CO\ molecules}{the\ number\ of\ incident\ photos} \times 100\%$$

For the CdS:

QE=

$$\frac{2 \times 0.98 \times 10^{-6} \times 6.02 \times 10^{23}}{5 \times 10^{-3} \times 4.91 \times 420 \times 10 \times 3600 \times 10^{-9} \Big/ 6.626 \times 10^{-34} \times 3 \times 10^{8}} \times \mathbf{100}$$

=0.063%

For the CdS@Co-bpy:

QE=

$$\frac{2 \times 4.7 \times 10^{-6} \times 6.02 \times 10^{23}}{5 \times 10^{-3} \times 4.91 \times 420 \times 10 \times 3600 \times 10^{-9} \Big/ 6.626 \times 10^{-34} \times 3 \times 10^{8}} \times \mathbf{100}$$

=0.302%

For the CdS@Co-bpy:

QE=

$$\frac{2\times 10.1\times 10^{-6}\times 6.02\times 10^{23}}{5\times 10^{-3}\times 4.91\times 420\times 10\times 3600\times 10^{-9} \Big/ 6.626\times 10^{-34}\times 3\times 10^{8}}\times \mathbf{100}$$

=0.65%

The turnover number (TON) calculations.

$$TON=\frac{the\ mole\ of\ evolved\ CO\ molecules}{the\ mole\ of\ the\ active\ sites}$$

$$=\frac{2350\times 10^{-6}\times 10\times 10^{-3}}{10\times 10^{-3}\times 0.0618 \Big/ 59}$$

=2.24

Photoreduction of CO_2. Photoreduction of CO_2 was conducted in a gas-closed system with a side irradiation Pyrex cell. The 300 W Xe lamp (Aulight CEL-HX, Beijing) equipped with a 420 nm cut-off filter was used as the light source. Typically, 10 mg of catalysts were dispersed in 5 mL of CH_3CN containing 1 mL of TEOA as sacrifice agent. The amounts of H_2 and CO gas were determined using a gas chromatograph (Agilent 4890D), and the liquid products were determined using a high performance liquid chromatography (LC-20AT). In the reuse experiments, the CdS/UiO-bpy/Co composites were washed by acetone for 6 times, and used for the next run directly after drying at 60 ℃ under vacuum. The isotope-labeled experiment was performed using $^{13}CO_2$ instead of $^{12}CO_2$, and the gas products were analyzed using gas chromatography-mass spectrometry (GC-MS, 7890A and 294 5975C, Agilent).

Action spectrum for the photocatalytic reaction. The system was similar with the above of photoreduction of CO_2, excepting that the 420 nm cut-off filter was replaced by the 420 nm, 475nm, 550nm, 600nm and 675nm optical band-pass filter, respectively.

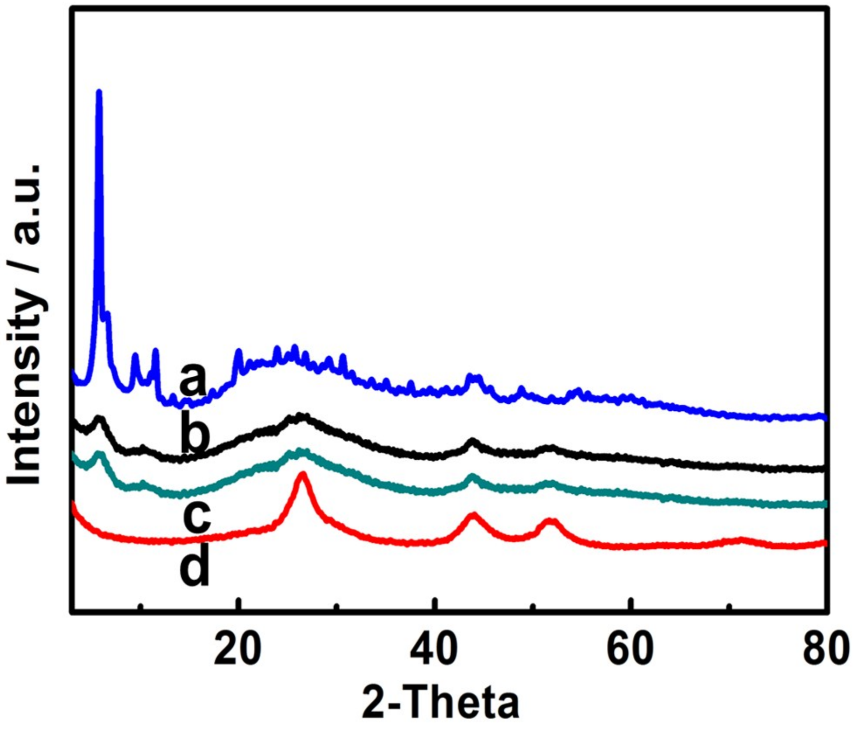

Figure S1. XRD patterns of UiO-bpy (a), CdS/UiO-bpy (b), CdS/UiO-bpy/Co(c) and CdS (d).

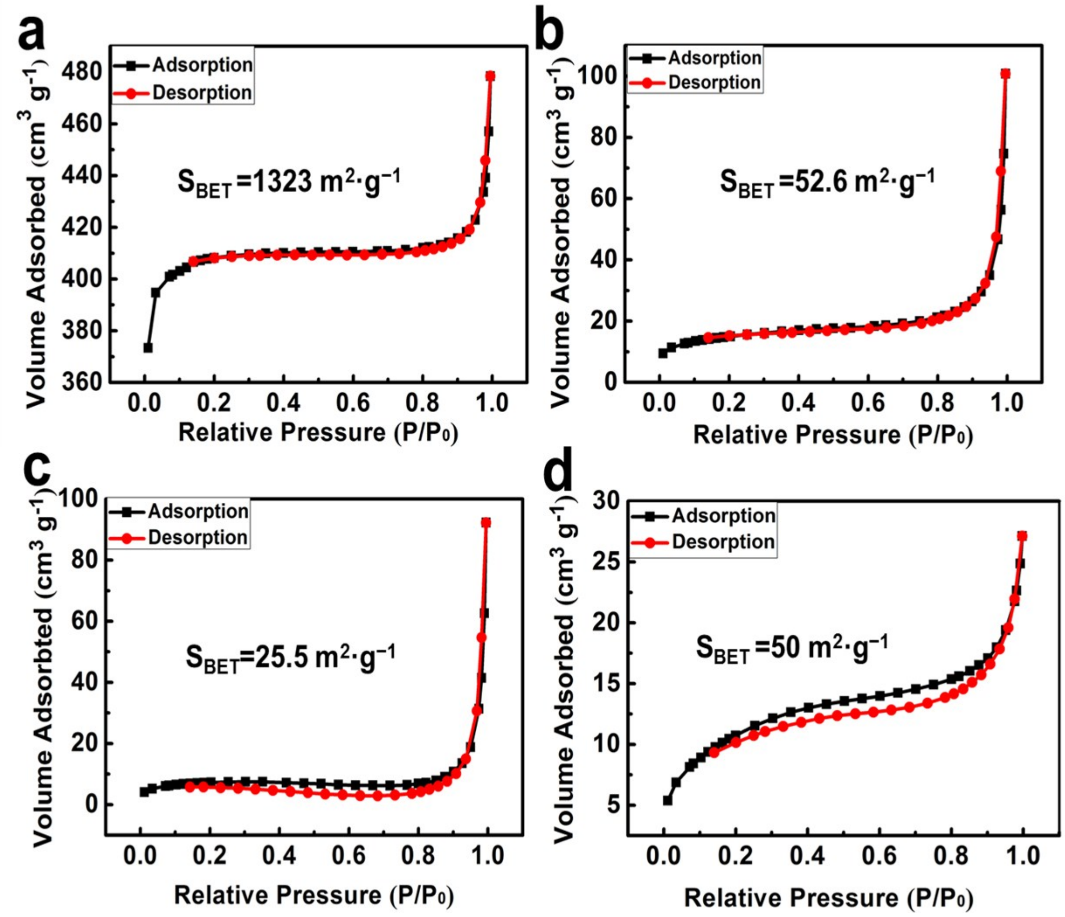

Figure S2 The adsorption-desorption isotherms of UiO-bpy(a), CdS/UiO-bpy(b), CdS/UiO-bpy/Co(c), and CdS(d). The BET surface areas (S_{BET}) are provided in the corresponding figures.

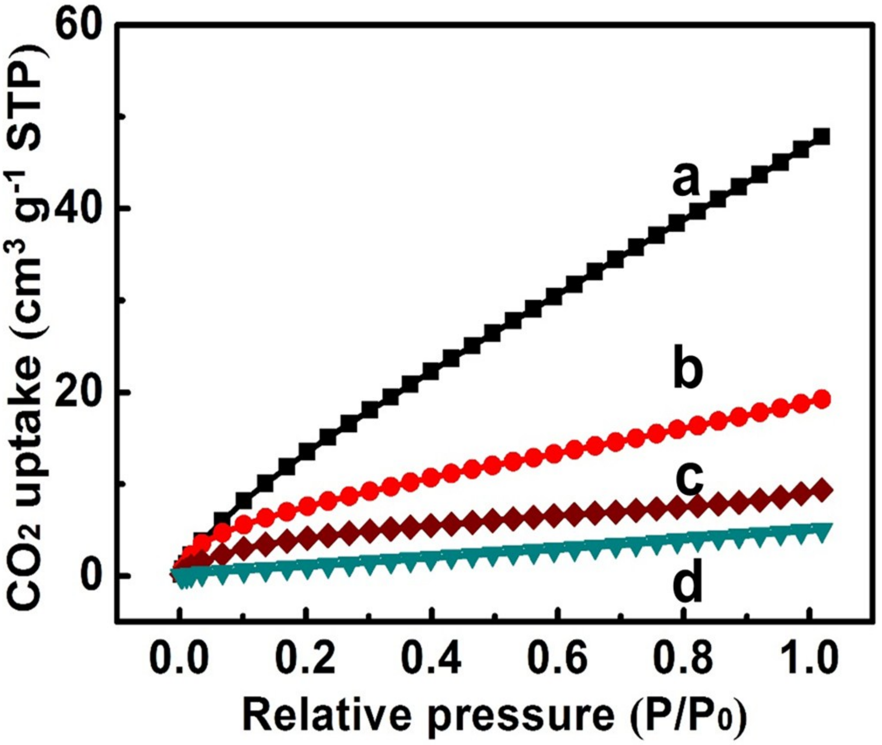

Figure S3 The CO_2 adsorption behaviors of UiO-bpy (a), CdS/UiO-bpy (b), CdS/UiO-bpy/Co (c), and CdS (d).

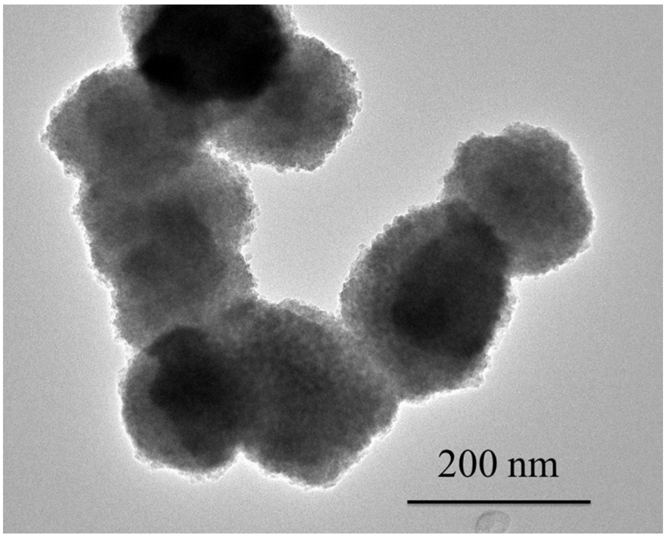

Figure S4. The TEM image of the pure CdS.

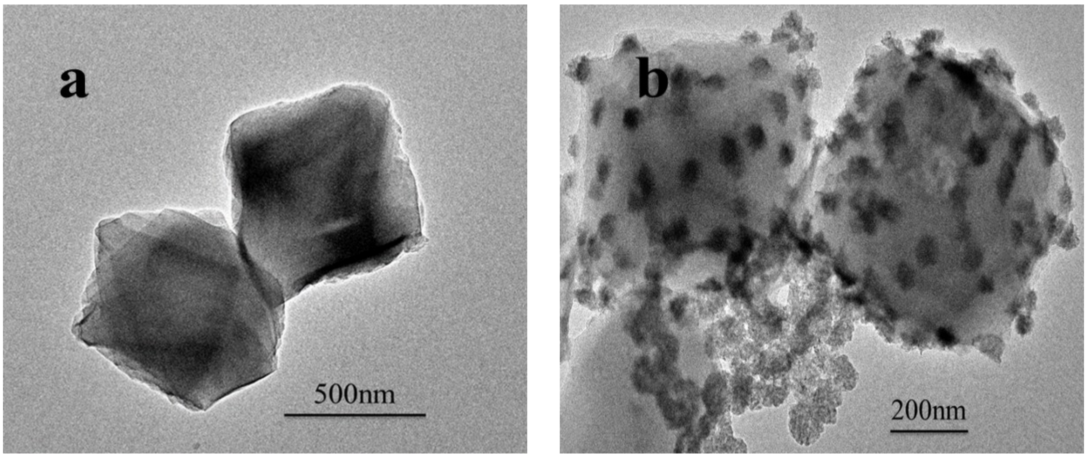

Figure S5. The TEM images of UiO-67 (a) and CdS/UiO-67 (b).

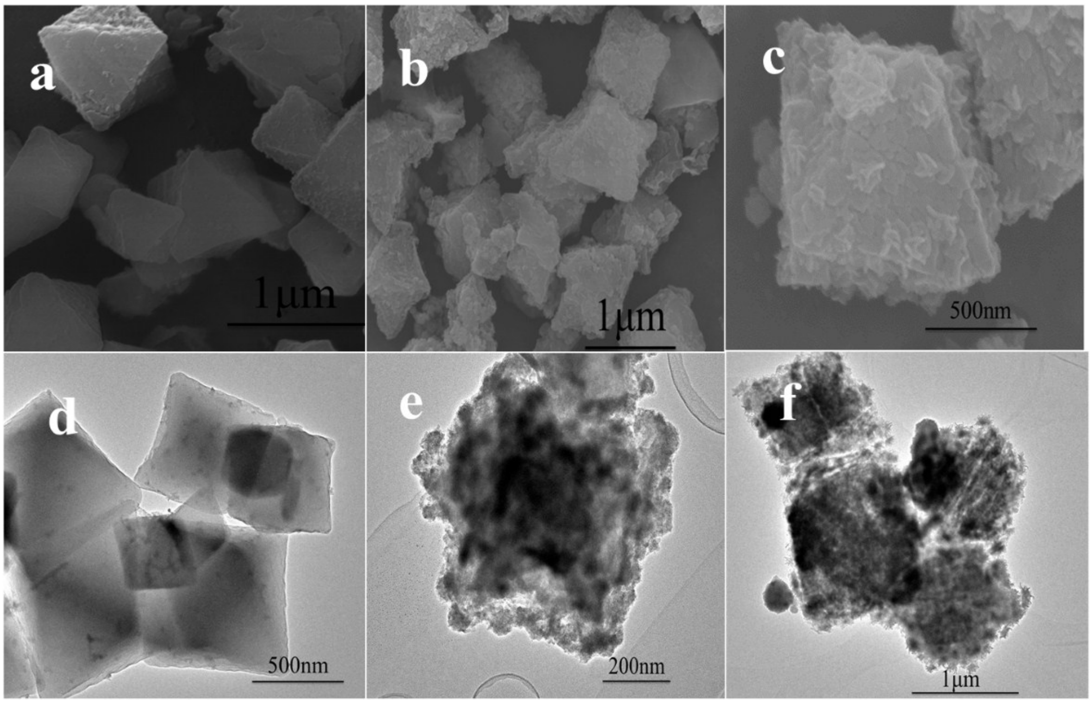

Figure S6. The SEM and TEM images of CdS/UiO-bpy with different contents of $Cd(CH_3COO)_2$: the cadmium acetate was 10 mg (a, d); the cadmium acetate was 60 mg (b, e); the cadmium acetate was 90 mg (c, f). The amount of UiO-bpy were all 300 mg.

We found that the amount of cadmium acetate had an important effect on the size of CdS. The UiO-bpy(300 mg) and cadmium acetate(10 mg) were added firstly into DMSO for 1 h under stirring, and then transformed to teflon kettle under 180°C for 12 h to form CdS/UiO-bpy. Only small amount of CdS nanoparticles were formed with the size of about 10 nm on the surface of UiO-bpy, as shown in Figures S6a and S6d. When the amount of cadmium acetate increased to 30 mg, although the number of CdS nanoparticles increased, the size of CdS nanoparticles on the UiO-bpy framework was still about 10 nm, as show in Figures 2B, 2C. As the amount of cadmium acetate increased to 60 mg and 90 mg, the size of CdS become bigger and covered a thick layer on the UiO-bpy, as shown in Figures S6b, 6c, 6e, and 6f. This is probably due to the fact that the growth rate of CdS was faster than the release rate of Cd^{2+} from the bridging ligands when the amount of cadmium acetate exceeds the critical value.

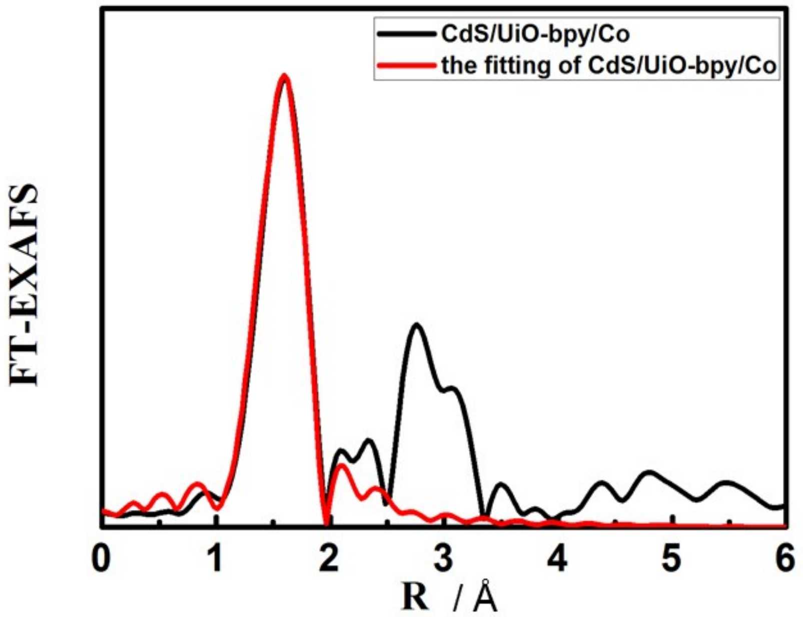

Figure S7. The EXAFS fitting curve of CdS/UiO-bpy/Co composites.

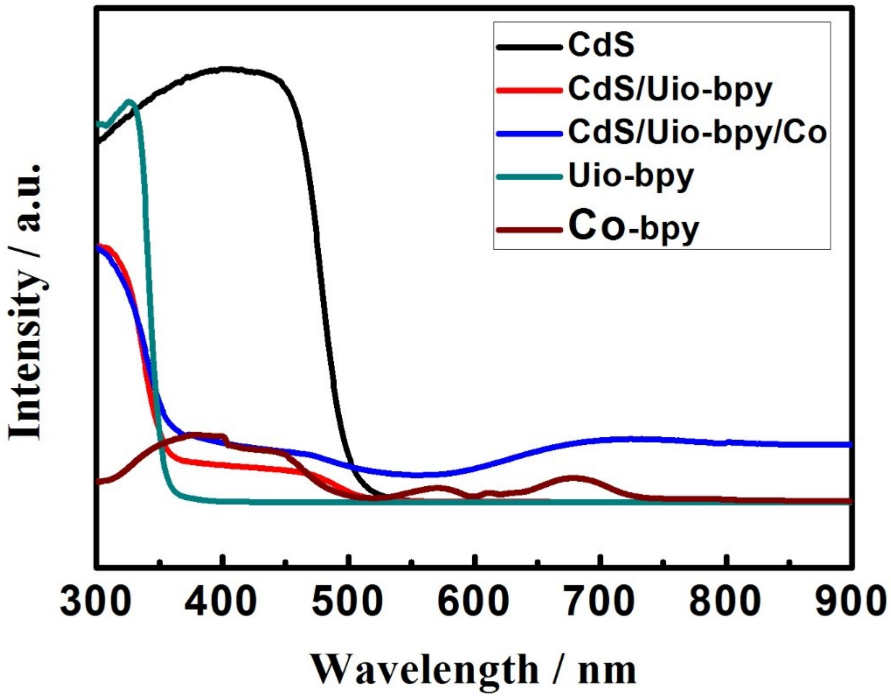

Figure S8. The diffuse reflectance UV/Vis spectra of CdS, UiO-bpy, CdS/UiO-bpy, CdS/UiO-bpy/Co composites and Co-bpy.

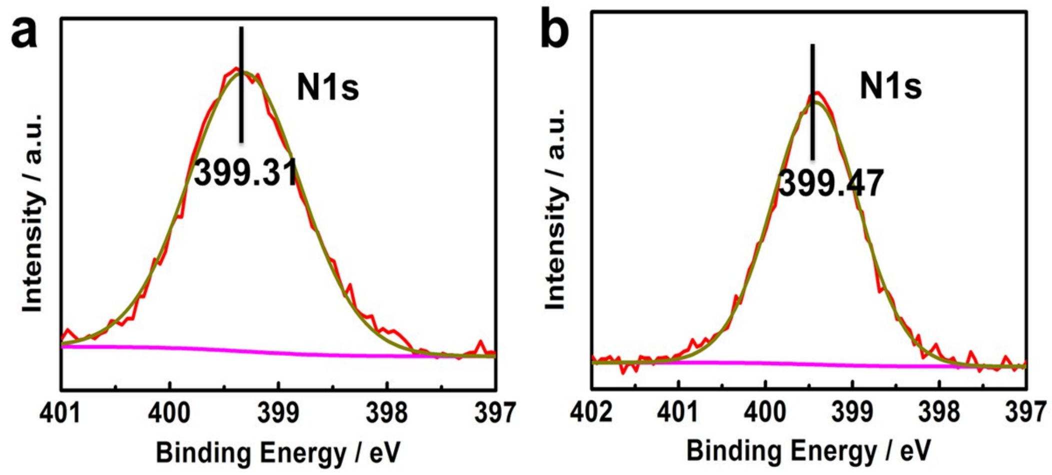

Figure S9. The XPS spectra of N 1s: the CdS/UiO-bpy (a) and the CdS/UiO-bpy/Co (b).

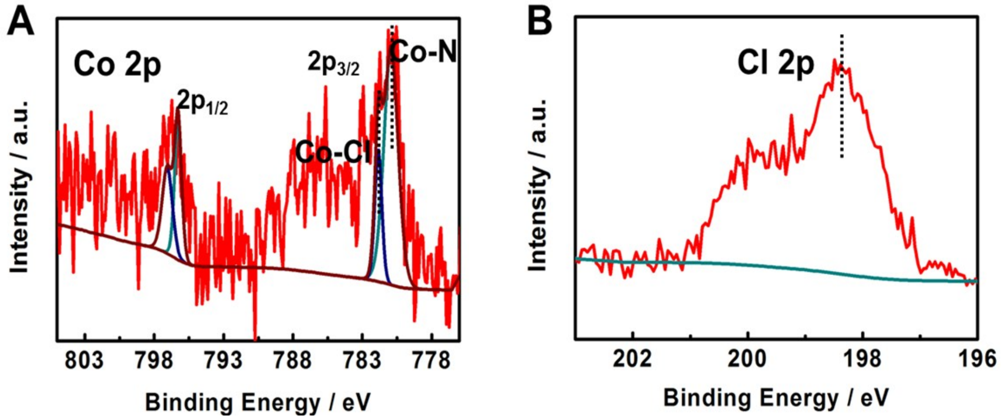

Figure S10. The XPS spectra of Co in CdS/UiO-bpy/Co (A); the XPS spectra of Cl in CdS/UiO-bpy/Co(B).

As shown in Figure S10A, we can find that the Co2p XPS spectrum of 780.8 eV and 781.8 eV can be assigned to the Co-N and Co-Cl, indicating that the Co was connected to the nitrogen and chlorine atoms simultaneously. These results were also certified by the XANES spectrum.

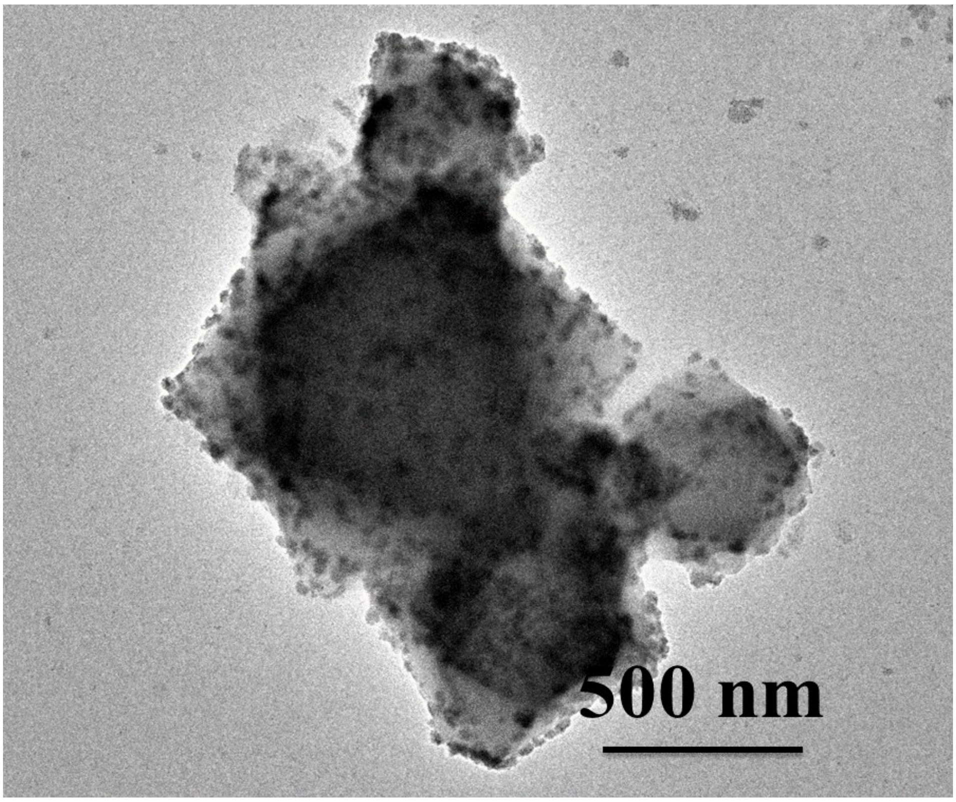

Figure S11. The TEM image of the CdS/UiO-bpy/Co after the five cycles.

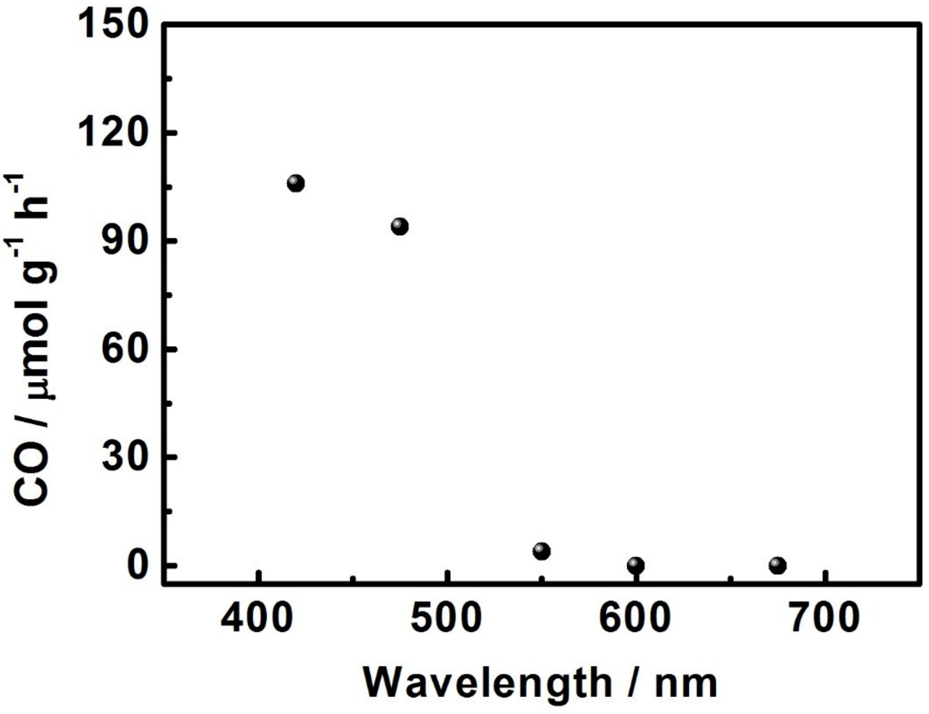

Figure S12. The action spectrum for the photocatalytic of CdS/UiO-bpy/Co composites.

As shown in Figure S12, the evolution rate of CO was 106, 94 and 4 μmol/g/h, respectively when the incident wavelength was 420, 475 and 550 nm. When the incident wavelength was 600 and 675 nm, the CO could not be detected. The performance of CdS/UiO-bpy/Co composites was consistent with the absorption behavior of CdS(Figure 4b). We can confirm that the reaction is induced by the light absorption of CdS.

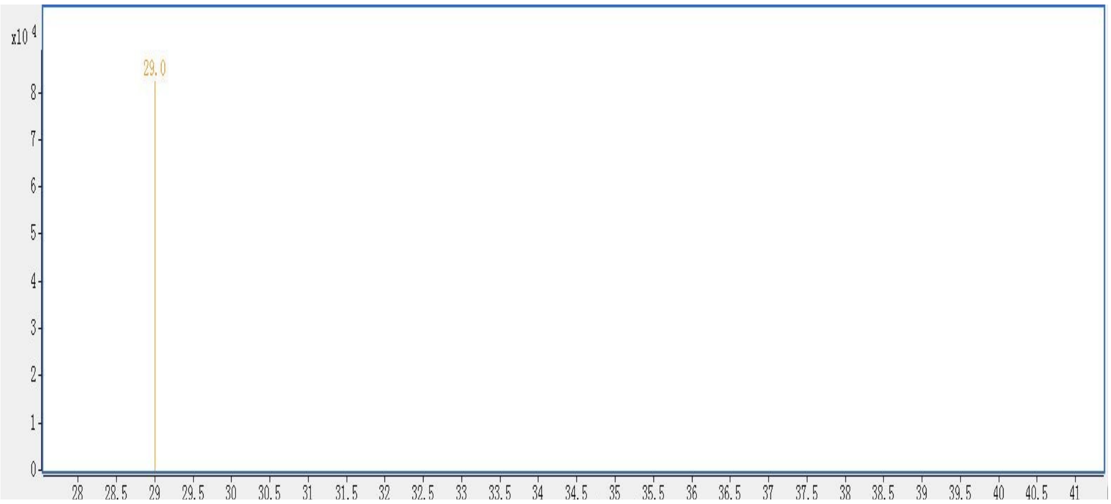

Figure S13 Mass spectrum of photogenerated product ^{13}CO (m/z=29) from photoreduction of $^{13}CO_2$ over the CdS/UiO-bpy/Co composites.

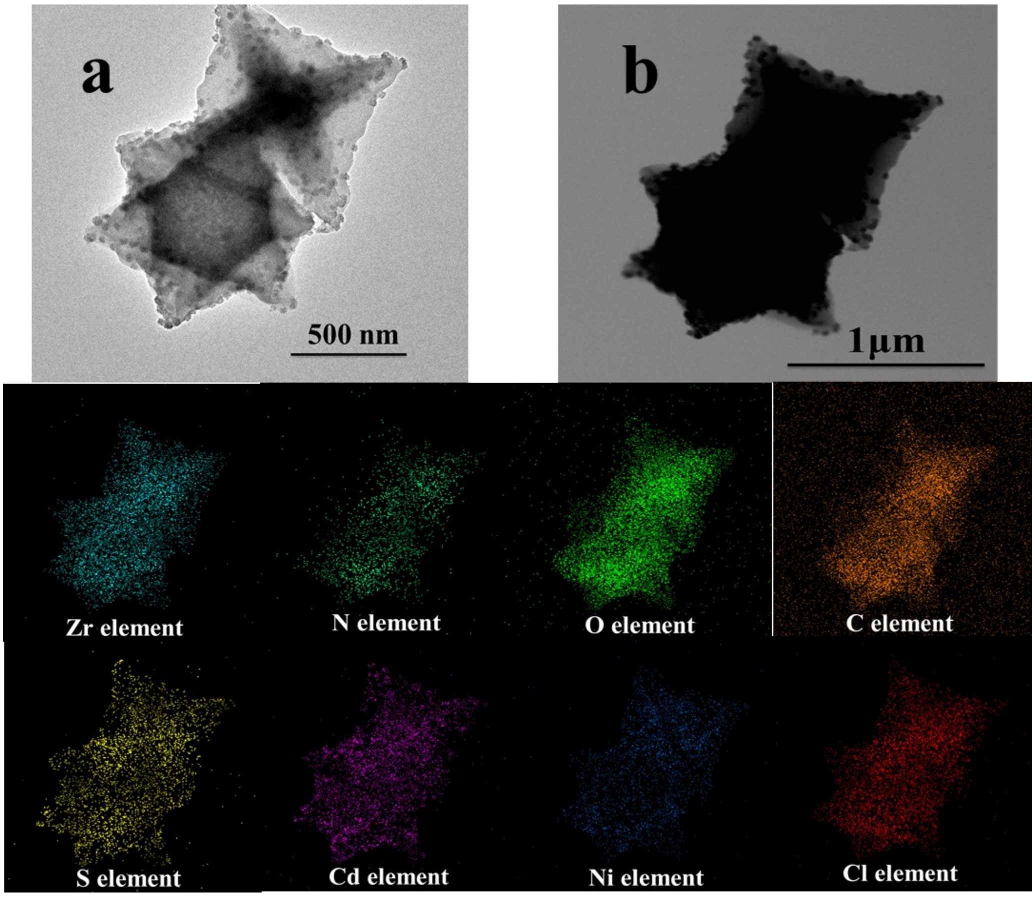

Figure S14. The TEM image of the CdS/UiO-bpy/Ni (a) and the EDX mapping of CdS/UiO-bpy/Ni(b).

Table S1. The contents of CdS and Co^{2+} (Ni^{2+}) in the CdS/UiO-bpy/Co (Ni) determined by ICP-AES method. 30 mg of $Cd(CH_3COO)_2{\cdot}2H_2O$ was used when preparing CdS/UiO-bpy.

Samples	Cd (wt%)	S (wt%)	Cd / S(atomic ratio)	Co or Ni (wt%)
**CdS/UiO-bpy/Co**	7.6	2.31	17 : 18	6.18
**CdS/UiO-bpy/Ni**	7.16	2.2	37 :39	6.01

Table S2. Structural parameters of CdS/UiO-bpy/Co extracted from the EXAFS fitting. (S_0^2=0.80)

Sample	Scattering pair	CN	R(Å)	$\sigma^2(10^{-3}Å^2)$	ΔE_0(eV)	R factor
CdS/UiO-bpy/Co	Co-N	2±0.9	1.95±0.02	9.6±1.7	5.6±1.4	0.0063
	Co-Cl	2.1±0.8	2.10±0.02	9.9±1.7	5.7±1.4	0.0074

S_0^2 is the amplitude reduction factor; CN is the coordination number; R is interatomic distance (the bond length between central atoms and surrounding coordination atoms); σ^2 is Debye-Waller factor (a measure of thermal and static disorder in absorber-scatterer distances); ΔE_0 is edge-energy shift (the difference between the zero kinetic energy value of the sample and that of the theoretical model). R factor is used to value the goodness of the fitting.

Table S3. Summary of the various photocatalytic systems employed for CO_2 reduction.

Catalyst	Co-catalyst / Sacrificial agent	Light source	Major products Rate	Ref.
CdS/UiO-bpy/Co	- /(MeCN/TEOA =5:1)	Visible light: 300 W Xe lamp with a cut-off filter (420<λ< 800 nm).	CO 235μmol $g^{-1}h^{-1}$(0.8MPa CO_2) 195μmol $g^{-1}h^{-1}$(0.1MPa CO_2)	This study
MOF-525-Co[a]	- /(MeCN/TEOA =4:1)	Visible light: 300 W Xe lamp with a cut-off filter (400<λ< 800 nm).	CO 201.6 μmol$g^{-1}h^{-1}$, CH_4 36.67 μmol $g^{-1}h^{-1}$	Zhang et.al. *Angew. Chem. Int. Ed.* 2016, 55, 14310.
PCN-222[b]	- /(MeCN/TEOA =4:1)	Visible light: 300 W Xe lamp with a cut-off filter (400<λ< 800 nm).	HCOOH 75μmol $g^{-1}h^{-1}$	Jiang *et.al J. Am.Chem. Soc.*2015,*137*, 13440.
Bismuth tungstate layers	- /H_2O	300 W Xe lamp with a standard AM1.5 filter.	CH_3OH 75 μmol $g^{-1}h^{-1}$	Xie *et.al Angew. Chem. Int. Ed.* 2015, *54*, 13971.
RuRu′ /C3N4[c]	Ag/ EDTA·2Na	Visible light: A 400 W high-pressure Hg lamp (SEN) (λ > 400 nm).	HCOOH 96 μmol $g^{-1}h^{-1}$	Maeda *et.al J. Am. Chem. Soc.* 2016, *138*, 5159.
UiO-66/C_3N_4[d]	- /(MeCN/TEOA =4:1)	Visible light: 300 W Xe lamp with a cut-off filter (400<λ< 800 nm).	CO 9.79 μmol $g^{-1}h^{-1}$	Ye *et. al Adv. Funct. Mater. 2015,* 25, 5360.
ZIF-9[e]	$[Ru(bpy)_3]Cl_2$ /acetonitrile: H_2O = 4: 1)	UV light: 300 W Xe lamp with a	CO 41.8μmol $g^{-1}h^{-1}$	*Wang et al. Angew. Chem. Int.*

		cut-off filter (λ < 400 nm).		*Ed. 2014, 53,* 1034
$ZnIn_2S_4$ Layers	- /H_2O	300 W Xe lamp with a standard AM1.5 filter.	CO 33.2μmol $g^{-1}h^{-1}$	Xie et.al. *J. Am. Chem. Soc.* 2017, 139, 7586
In_2S_3−$CdIn_2S_4$	bipyridine (bpy), $CoCl_2$/ acetonitrile: H_2O:TEOA = 3: 2:1)	Visible light: 300 W Xe lamp with a cut-off filter (400<λ< 800 nm).	CO 825μmol $g^{-1}h^{-1}$	Wang et.al. *J. Am. Chem. Soc.* 2017, 139, 17305
a, b Porphyrin based MOF; c Ru(II) binuclear complex; d Zirconium containing MOF; e ZIF=zeolitic imidazolate framework.				

S1 B. An, J. Zhang, K. Cheng, P. Ji, C. Wang, W. Lin, *J. Am. Chem. Soc.* **2017**, *139*, 3834-3840.
